# Availability of Medications for Opioid Use Disorder in Community Mental Health Facilities

**DOI:** 10.1001/jamanetworkopen.2024.17545

**Published:** 2024-06-18

**Authors:** Jonathan Cantor, Beth Ann Griffin, Barbara Levitan, Sapna J. Mendon-Plasek, Bradley D. Stein, Sarah B. Hunter, Allison J. Ober

**Affiliations:** 1 RAND, Santa Monica, California; 2 RAND, Arlington, Virginia; 3 RAND, Boston, Massachusetts; 4 RAND, Pittsburgh, Pennsylvania

## Abstract

**Importance:**

Medications for opioid use disorder (MOUD) are an effective but underutilized treatment. Opioid use disorder prevalence is high among people receiving treatment in community outpatient mental health treatment facilities (MHTFs), but MHTFs are understudied as an MOUD access point.

**Objective:**

To quantify availability of MOUD at community outpatient MHTFs in high-burden states as well as characteristics associated with offering MOUD.

**Design, Setting, and Participants:**

This cross-sectional study performed a phone survey between April and July 2023 among a representative sample of community outpatient MHTFs within 20 states most affected by the opioid crisis, including all Certified Community Behavioral Health Centers (CCBHCs). Participants were staff at 450 surveyed community outpatient MHTFs in 20 states in the US.

**Main Outcomes and Measures:**

MOUD availability. A multivariable logistic regression was fit to assess associations of facility, county, and state-level characteristics with offering MOUD.

**Results:**

Surveys with staff from 450 community outpatient MHTFs (152 CCBHCs and 298 non-CCBHCs) in 20 states were analyzed. Weighted estimates found that 34% (95% CI, 29%-39%) of MHTFs offered MOUD in these states. Facility-level factors associated with increased odds of offering MOUD were: self-reporting being a CCBHC (odds ratio [OR], 2.11 [95% CI, 1.08-4.11]), providing integrated mental and substance use disorder treatment (OR, 5.21 [95% CI, 2.44-11.14), having a specialized treatment program for clients with co-occurring mental and substance use disorders (OR, 2.25 [95% CI, 1.14-4.43), offering housing services (OR, 2.54 [95% CI, 1.43-4.51]), and laboratory testing (OR, 2.15 [95% CI, 1.12-4.12]). Facilities that accepted state-financed health insurance plans other than Medicaid as a form of payment had increased odds of offering MOUD (OR, 1.95 [95% CI, 1.01-3.76]) and facilities that accepted state mental health agency funds had reduced odds (OR, 0.43 [95% CI, 0.19-0.99]).

**Conclusions and Relevance:**

In this study of 450 community outpatient MHTFs in 20 high-burden states, approximately one-third offered MOUD. These results suggest that further study is needed to report MOUD uptake, either through increased prescribing at all clinics or through effective referral models.

## Key Points

**Question:**

What is the availability of medications for opioid use disorder (MOUD) in a representative sample of community outpatient mental health treatment facilities in high-burden states due to the opioid crisis?

**Findings:**

In this cross-sectional study of 450 community outpatient mental health treatment facilities in 20 states, 34% of clinics offered MOUD, with 51% of Certified Community Behavioral Health Clinics (CCBHCs) and 33% of non-CCBHCs offering these medications.

**Meaning:**

Despite high rates of opioid use disorder among people with co-occurring mental health disorders, only a third of community outpatient mental health treatment facilities in high-need states offer MOUD, indicating the need for improved scaling efforts.

## Teaser

This cross-sectional study examines the availability of medications for opioid use disorder (MOUD) at community outpatient mental health treatment facilities in 20 US high-burden states.

## Introduction

The opioid crisis is an ongoing and urgent public health emergency, with 80 401 opioid overdose deaths in 2021,^1^ despite effective, life-saving medications. Collectively referred to as medications for opioid use disorder (MOUD), these medications—buprenorphine, methadone, and naltrexone—have become the standard for opioid use disorder (OUD) recovery.^2^ MOUD use has been associated with reductions in health care use, including both inpatient and outpatient care, as well as decreased overdose mortality.^3,4,5,6^

Despite its effectiveness and the treatment need, MOUD remains underused.^7^ A recent study found that nearly 90% of those with OUD did not receive MOUD.^8^ Barriers to MOUD access persist despite ongoing efforts to improve capacity, including policy changes.^9,10^ For example, Medicaid expansions through the Affordable Care Act increased the number of individuals who could receive Medicaid reimbursed substance use disorder (SUD) treatment services. Despite these changes, MOUD are still underused.^11^

MOUD access is further complicated by the many individuals with co-occurring OUD and mental health disorders (COD); an estimated 25% of adults with OUD having a co-occurring serious mental illness.^12^ Complexities in navigating multiple systems (eg, primary care, specialty SUD care, mental health care) pose additional barriers for adults with COD,^13^ requiring improved understanding of how to increase access and treatment utilization.

Because individuals with COD are more likely to receive treatment for mental health than an SUD, and given the high OUD prevalence in community mental health treatment facilities (MHTFs),^14^ MHTFs are a potentially important MOUD access point. Increasing MOUD delivery in physical health care settings^15^ has been a priority, with less attention to increasing delivery in MHTFs,^16^ which has the potential to improve access to the more than 13 million individuals with COD who receive care in such settings annually.^17^

Certified Community Behavioral Health Clinics (CCBHCs), an important subset of MHTFs, are required to provide a range of streamlined behavioral health care services.^18^ This includes SUD services,^15^ although CCBHCs are not required to provide MOUD, but may instead have an arrangement with a health care organization that provides MOUD. Despite the opportunity for CCBHCs to expand MOUD availability within MHTFs, a recent study found that only 34% of CCBHCs offered MOUD.^16^

To provide an accurate, representative picture of MOUD availability in community outpatient MHTFs, this study examines self-reported MOUD provision among a representative sample of community outpatient MHTFs in 20 US states with the highest needs in 2020, including all CCBHCs within those states. We report the prevalence of MOUD availability as well as facility, geographic, and state policy factors associated with whether MOUD is offered.

## Methods

The RAND Survey Research Group called community outpatient MHTFs in 20 states between April 12 and July 31, 2023. They administered a standardized, 10-item survey (eAppendix in Supplement 1) containing items from existing treatment facility surveys^16,19,20^ and locally developed items to identify type and extent of MOUD provision and related services. We inquired about whether MOUD was offered, whether it was offered on-site or at a different facility within or outside of the same organization, and which medications were offered. No compensation was given. Respondents are asked to give permission to include select survey responses in the Substance Abuse and Mental Health Services Administration’s Behavioral Health Treatment Locator.^22^ This cross-sectional study was approved by the RAND institutional review board and followed the Strengthening the Reporting of Observational Studies in Epidemiology (STROBE) reporting guideline for a cross-sectional study.

### Sampling Design

The sampling frame was derived from community outpatient MHTFs responding to the National Substance Use and Mental Health Services Survey and who agreed to be listed in the Substance Abuse and Mental Health Services Administration Behavioral Health Treatment Locator. The locator is considered to be the most comprehensive resource for mental health treatment.^22^ To better understand MOUD availability in states most affected by the opioid crisis, we restricted the survey to facilities located in 20 high-burden states defined as states that were any of the following: (1) in the top quartile for drug overdose deaths per 100 000 people in 2020; (2) in the top quartile for an increase in drug overdose death rates between 2019 and 2020 (if not included in the prior category); or (3) in the top quartile for the total number of drug overdose deaths (if >3000), if not included in either previous category. Included states were Arizona, California, Connecticut, Delaware, Florida, Indiana, Kentucky, Maine, Maryland, New Mexico, New York, Ohio, Pennsylvania, Rhode Island, South Carolina, Tennessee, Texas, Vermont, West Virginia, and Wyoming.

To focus on community outpatient MHTFs serving adults potentially in need of MOUD, we identified facilities that accepted public funding (ie, Medicaid or state block grants) as reported in the National Substance Use and Mental Health Services Survey. Of the 4251 outpatient facilities in the 20 states, 3906 met these criteria (91.88%).

The National Substance Use and Mental Health Services Survey is administered to all MHTFs in the US that are licensed, certified, or approved by state mental health agencies to provide mental health treatment. The RAND Mental health and Addiction Treatment Tracking Repository regularly downloads and stores data from the Behavioral Health Treatment Locator to track the availability of treatment services over time. The data include information about the facility, including funding sources, related ancillary services (eg, laboratory testing, housing recovery services), program offerings (eg, special or integrated programs) and policies, including whether the facility is a CCBHC. We selected our survey sample from the repository’s data downloaded on March 28, 2023.

Our goal was to create a representative sample capturing the rate of offering MOUD by both CCBHCs and non-CCBHCs. Given the low number of CCBHCs in selected states (approximately 6%), we sampled all facilities self-reporting as CCBHCs. Among non-CCBHCs, we selected a proportionate random sample of facilities by state to ensure sampled facilities’ distribution was representative of all facilities in those states, equating to performing a stratified random sampling of non-CCBHCs by state for selected states.

### Nonresponse and Survey Weighting

To facilitate generalizing findings to the population of community outpatient MHTFs responding to the National Substance Use and Mental Health Services Survey in the selected states (3906 facilities [213 CCBHCs and 3693 non-CCBHCs]), we calculated a set of analytic weights for our final sample of 450 responding facilities that accounted for both sampling design and nonresponse. The sampling weight for each facility was equal to the inverse of the probability of selection into the sample and accounted for the proportionate sampling design for non-CCBHCs as well as the oversampling of CCBHCs. The sampling weights were then multiplied by nonresponse weights to further account for loss to follow-up among the nonresponding facilities. Nonresponse weights were set equal to the inverse probability of response by state for non-CCBHCS and overall for CCBHCs. The sampling and nonresponse weights were then multiplied together to obtain the final analytic weights.

### Statistical Analysis

We first assessed the final sample of responding facilities’ generalizability by comparing them to all community outpatient MHTFs in the selected states on key facility characteristics (eg, funding source, types of services offered). Next, we examined unadjusted differences between facilities that did and did not report offering MOUD in those states. We used standardized mean differences (SMDs or effect sizes) to denote meaningful differences between groups. SMDs take the mean difference on a given facility-level characteristic (eg, funding source) between groups being compared and then divide by the standard deviation of the characteristic. In assessing the generalizability of our sample, we considered anything greater than 0.1 to be indicative of a meaningful difference.^23,24,25^ When comparing facilities that did and did not report offering MOUD, we assigned ranges of differences in SMD as small (0-0.3), medium (0.3-0.6), or large (>0.6). Additionally, we estimated a multivariate logistic regression examining adjusted associations between facility-, county-, and state-level characteristics and the likelihood of a facility reporting that it offered MOUD. Most variables used in this assessment were from either RAND Mental health and Addiction Treatment Tracking Repository or from our survey, with 2 exceptions: State Medicaid Expansion status from the Kaiser Family Foundation^26^ and county metropolitan status from the National Center for Health Statistics.^27^ Our approach for performing variable selection and checking for multicollinearity between facility-level characteristics is available in the eAppendix in Supplement 1. Finally, we analyzed survey frequencies for facilities that did and did not offer MOUD. All analyses used the final analytic weights composed of the sampling and nonresponse weights. A 2-sided 5% level was used for statistical significance. All analyses were completed using Stata version 18 (StataCorp) as well as R version 4.3.2 (R Project for Statistical Computing).^21^

## Results

We contacted 642 community outpatient MHTFs. Table 1 provides facility-level characteristics of 3906 targeted facilities, 642 sampled facilities, and the 450 facilities responding to the survey (70.1%), including details on the facilities offering and not offering MOUD. We found no evidence of differences between sampled and targeted facilities, suggesting the sampling design was well executed. We also found no evidence of differences between the responding and targeted facilities after adjusting for the study analytic weights, suggesting our final sample is still representative of the target population of facilities. Details about nonresponding facilities are included in the eAppendix in Supplement 1.

**Table 1.** Descriptive Statistics of Target Population, Sampled Facilities, Respondents, and Facilities That Did and Did Not Offer MOUD^a^

Characteristics	Facilities, No. (weighted %)				
	Target population (N = 3906 facilities)	Sampled sites (N = 642 facilities)	Respondents (n = 450 facilities)	Respondents (n = 450 facilities)	
				Offered MOUD (n = 178 facilities)	Did not offer MOUD (n = 272 facilities)
Certified Community Behavioral Health Clinics	213 (6)	213 (6)	152 (6)	77 (9)	75 (4)
Payment/insurance/funding accepted					
State-financed health insurance plan other than Medicaid	2291 (59)	420 (57)	304 (59)	129 (65)	175 (56)^b^
State mental health agency (or equivalent) funds	1985 (51)	369 (50)	278 (54)	107 (45)	171 (58)
Community Service Block Grants	815 (21)	183 (19)	144 (22)	60 (22)	84 (22)
Community Mental Health Block Grants	1329 (34)	267 (32)	202 (34)	83 (31)	119 (36)
County or local government funds	1694 (43)	293 (40)	208 (41)	74 (35)	134 (44)^b^
State corrections or juvenile justice funds	1086 (28)	242 (27)	181 (29)	75 (26)	106 (30)
State education agency funds	587 (15)	97 (14)	76 (15)	29 (13)	47 (16)
State welfare or child and family services funds	1600 (41)	303 (41)	224 (42)	90 (36)	134 (45)
Federal military insurance (eg, TRICARE)	1772 (45)	350 (50)	260 (52)^c^	120 (63)	140 (46)^b^
US Department of VA funds	792 (20)	183 (24)	136 (25)^c^	69 (33)	67 (20)^b^
Special programs/groups offered					
Clients with co-occurring mental and substance use disorders	2,290 (59)	424 (60)	320 (65)^c^	144 (77)	176 (59)^b^
Persons with traumatic brain injury	439 (11)	78 (14)	67 (17)^c^	35 (24)	32 (13)^b^
Clients with HIV or AIDS	646 (17)	110 (19)	88 (21)^c^	49 (30)	39 (16)^b^
Integrated mental and substance use disorder treatment	2594 (66)	471 (68)	343 (71)^c^	159 (90)	184 (61)^b^
Housing services	962 (25)	192 (25)	151 (28)	79 (40)	72 (22)^b^
Laboratory testing	1139 (29)	197 (28)	131 (26)	70 (41)	61 (19)^b^
Facility operation					
Private nonprofit organization	2514 (64)	423 (61)	302 (63)	125 (59)	177 (65)
Private for-profit organization	699 (18)	98 (15)	66 (19)	23 (19)	43 (19)^b^
Government	693 (18)	121 (19)	82 (18)	30 (22)	52 (16)
Vaping permitted in designated area	1414 (36)	228 (39)	169 (40)	67 (42)	102 (39)
Suicide prevention services	2754 (71)	481 (71)	352 (74)	141 (75)	211 (74)
Smoking permitted in designated area	1578 (40)	250 (43)	186 (45)	73 (46)	113 (44)
Federally Qualified Health Center	447 (11)	80 (14)	59 (14)	33 (21)	26 (10)
Urban	2949 (76)	452 (75)	299 (72)	120 (70)	179 (73)
State Medicaid expanded	3170 (81)	478 (81)	339 (82)	149 (86)	190 (79)
Census region					
Midwest	722 (19)	106 (19)	75 (18)	41 (26)	34 (14)^b^
Northeast	1056 (28)	176 (27)	116 (26)	52 (26)	64 (27)
South	1342 (34)	266 (35)	192 (35)	64 (32)	128 (38)
West	786 (20)	94 (20)	67 (20)	21 (16)	46 (20)

Abbreviation: MOUD, medications for opioid use disorder.

^a^ Three standardized mean differences (SMDs) were calculated. The first compared the sampled population with the target population, although no SMD greater than 0.1 was found, so no superscript was needed. The second compared respondents with the target population. The third compared facilities that did and did not offer MOUD.

^b^ SMD greater than 0.1.

^c^ SMD greater than or equal to 0.3.

When examining unadjusted differences between facilities offering and not offering MOUD, we found a few meaningful unadjusted differences. Facilities offering MOUD were more likely to accept federal military insurance (eg, TRICARE: SMD, 0.34), offer housing services (SMD, 0.38) and be located in the Midwest (SMD, 0.30). They were also more likely to have a specialized treatment program for persons with TBI, HIV, or AIDs, and individuals with co-occurring mental and SUDs (SMDs ranged from 0.30 to 0.42), to conduct laboratory testing (SMD, 0.49), and to be FQHCs (SMD, 0.32). Private nonprofit organizations were less likely to provide MOUD compared with private for-profit organizations.

Our logistic regression found that facility-level factors associated with increased odds of offering MOUD after adjusting jointly for all key covariates were (1) being a CCBHC (OR, 2.11 [95% CI, 1.08-4.11]), (2) providing integrated mental and SUD treatment (OR, 5.21 [95% CI, 2.44-11.14]), (3) having a specialized treatment program for clients with co-occurring mental and SUDs (OR, 2.25 [95% CI, 1.14-4.43]), (4) offering housing services (OR, 2.54 [95% CI, 1.43-4.51]), and (5) offering laboratory testing (OR, 2.15 [95% CI, 1.12-4.12]) (Table 2). Facilities accepting state-financed health insurance plans other than Medicaid as a form of payment also had increased odds of offering MOUD (OR, 1.95 [95% CI, 1.01-3.76]) while facilities accepting state mental health agency funds had reduced odds (OR, 0.43 [95% CI, 0.19-0.99]). Approximately one-third of facilities (34% [95% CI, 29%-39%]) offered MOUD (Figure); the rate was higher in CCBHCs (51% [95% CI, 43%-59%]) than non-CCBHCs (33% [95% CI, 28%-39%]).

**Table 2.** Logistic Regression Estimating MOUD Availability^a^

Characteristic	MOUD offered at facility, OR (95% CI)
Certified Community Behavioral Health Clinic	2.11 (1.08-4.11)
Payment/insurance/funding accepted	
State-financed health insurance plan other than Medicaid	1.95 (1.01-3.76)
State mental health agency (or equivalent) funds	0.43 (0.19-0.99)
Community mental health block grants	1.03 (0.46-2.29)
Community service block grants	0.63 (0.26-1.57)
County or local government funds	0.96 (0.47-1.99)
State corrections or juvenile justice funds	0.74 (0.32-1.73)
State education agency funds	0.80 (0.30-2.13)
State welfare or child and family services funds	0.78 (0.37-1.67)
Federal military insurance (eg, TRICARE)	1.61 (0.80-3.24)
US Department of VA funds	1.95 (0.88-4.31)
Special programs/groups offered	
Clients with co-occurring mental and substance use disorders	2.25 (1.14-4.43)
Persons with traumatic brain injury	1.02 (0.39-2.67)
Clients with HIV or AIDS	0.91 (0.40-2.04)
Integrated mental and substance use disorder treatment	5.21 (2.44-11.14)
Housing services	2.54 (1.43-4.51)
Laboratory testing	2.15 (1.12-4.12)
Facility operation (eg, private, public)	
Private nonprofit organization	0.91 (0.42-1.96)
Private for-profit organization	0.94 (0.33-2.67)
Vaping permitted in designated area	1.84 (0.39-8.59)
Suicide prevention services	0.56 (0.26-1.23)
Smoking permitted in designated area	0.83 (0.18-3.85)
Federally Qualified Health Center	1.90 (0.87-4.14)
Metropolitan (vs not)	0.99 (0.53-1.84)
State Medicaid expanded (vs not)	2.22 (0.77-6.43)
Census region (baseline is South)	
Midwest	1.67 (0.68-4.06)
Northeast	1.05 (0.45-2.49)
West	0.49 (0.19-1.27)

Abbreviations: MOUD, medications for opioid use disorder; OR, odds ratio; VA, Veterans Affairs.

^a^ Outcome measure is from the question: “Do you offer medication at this location (at this address) for people who have an opioid use disorder alongside a mental health disorder? By medication I mean buprenorphine, such as Suboxone, or methadone or naltrexone, such as Vivitrol.” Sampling weights were used when estimating the regression.

**Figure.**
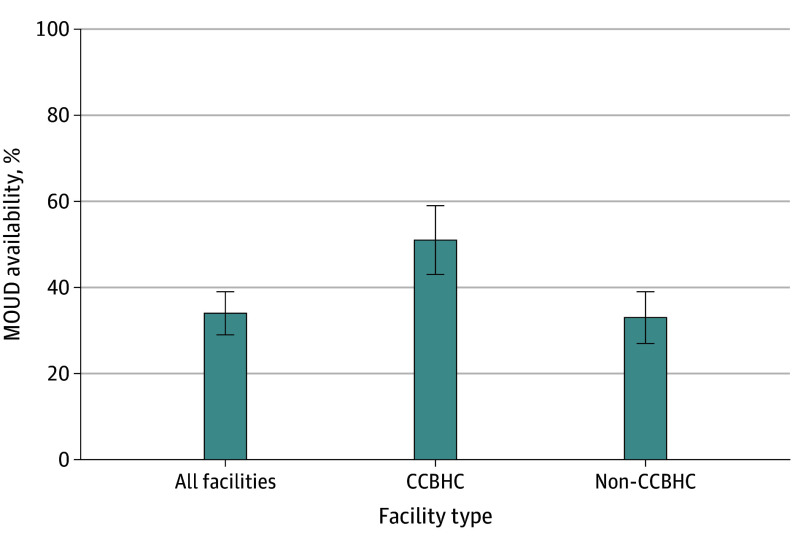
MOUD Availability by CCBHC Status CCBHC indicates Certified Community Behavioral Health Clinic; MOUD, medications for opioid use disorder.

Table 3 shows weighted survey responses for MHTFs that reported offering MOUD. The majority (84% [95% CI, 77%-91%]) offered buprenorphine with 96% (95% CI, 91%-100%) offering oral/sublingual forms, 43% (95% CI, 34%-52%) offering injectable buprenorphine, and fewer than 1% (95% CI, −1% to 4%) offering subcutaneous implants. Additionally, 70% (95% CI, 62%-79%) of MHTFs offered naltrexone and 14% (95% CI, 8%-21%) offered methadone. Approximately half (47% [95% CI, 38%-56%]) reported screening patients for OUD, however, 20% (95% CI, 12%-27%) reported not knowing the answer to this survey question. Most (70% [95% CI, 61%-78%]) reported patients paying for MOUD using private insurance, 84% (95% CI, 78%-91%) using Medicaid, 67% (95% CI, 59% to 76%) using Medicare, and 75% (95% CI, 66%-83%) paying out-of-pocket. Regarding services offered to individuals with COD, 91% (95% CI, 86%-96%) of facilities reported offering individual counseling, 78% (95% CI, 71%-86%) group counseling or therapy, 82% (95% CI, 75%-88%) telehealth for OUD, and 60% (95% CI, 51%-69%) reported offering telehealth for MOUD specifically. More than three-quarters of facilities offered services in languages other than English. The mean number of days patients needed to wait for a MOUD appointment was 12.7 (95% CI, 9.4-15.9) days.

**Table 3.** Survey Responses for Facilities That Offer MOUD (n = 178)

Questions	All facilities, % (95% CI)^a^
Which medications for opioid use disorder does the clinic offer? (n = 178)	
Buprenorphine	84.01 (77.39 to 90.63)
Naltrexone	70.24 (61.79 to 78.69)
Methadone	14.48 (8.01 to 20.95)
For clinics offering buprenorphine, what types does the clinic offer? (n = 148)	
Oral or sublingual	95.55 (91.22 to 99.87)
Injectable	42.52 (32.63 to 52.41)
Implant	1.29 (−1.25 to 3.83)
Do not know	2.10 (−0.82 to 5.02)
Mean No. of days until appointment for MOUD (95% CI) (n = 178)	12.67 (9.42 to 15.93)
How would someone pay for their (buprenorphine/methadone/naltrexone) here? (n = 178)	
Medicaid	84.40 (77.53 to 91.28)
Out-of-pocket (self-pay/private pay)	74.54 (66.44 to 82.65)
Private insurance	69.77 (61.22 to 78.31)
Medicare	67.27 (58.63 to 75.91)
Do not know	2.08 (−0.51 to 4.68)
Does this clinic specifically screen all patients for opioid use disorder at intake? (n = 178)	
Yes	46.86 (37.77 to 55.94)
No	33.23 (24.61 to 41.85)
Do not know	19.91 (12.42 to 27.41)
What services besides medication are offered at this clinic for people with a co-occurring mental health and opioid use disorder who are on medication for opioid use? (n = 178)	
Individual counseling for substance use disorder	91.01 (85.85 to 96.16)
Telehealth (audio and/or video) for counseling/therapy visits for opioid use disorder	81.55 (74.66 to 88.44)
Group counseling/therapy for substance use disorder	78.02 (70.54 to 85.49)
Telehealth (audio and/or video) for visits related to medications for opioid use disorder	59.60 (50.69 to 68.51)
Are services available in languages other than English at this clinic? (n = 178)	
Yes	77.63 (69.85 to 85.41)
No	18.25 (10.97 to 25.54)
Do not know	4.12 (0.54 to 7.96)

^a^ Sampling weights were used when calculating these frequencies.

In Table 4 we report weighted survey responses among facilities not offering MOUD. One-third of these facilities (33% [95% CI, 27%-40%]) reported screening all patients for OUD, however, 23% (95% CI, 17%-29%) reported not knowing whether the facility did this. Approximately 7% (95% CI, 4%-11%) of these facilities planned to offer MOUD in the future, but 39% (95% CI, 32%-46%) reported not knowing about future offerings of MOUD. There was not a statistically significant difference to the question on planning to start providing MOUD by CCBHC status. Notably, 87% (95% CI, 83%-92%) of these facilities refer patients elsewhere for MOUD treatment. Among these facilities, 38% (95% CI, 31%-45%) referred patients to other facilities within the same organization, 48% (95% CI, 36%-60%) referred patients to a mental health clinic, 67% (95% CI, 56%-78%) to an SUD treatment facility or opioid treatment program, and 9% (95% CI, 2%-16%) to a health clinic. In contrast to facilities referring patients for MOUD to external facilities, 77% (95% CI, 69%-85%) referred patients to an SUD treatment facility and 33% (95% CI, 25%-42%) to a health clinic. Only approximately 22% (95% CI, 12%-32%) reported referring patients to receive telehealth for MOUD. Among facilities referring elsewhere, 60% (95% CI, 53%-64%) reported OUD counseling or group therapy was available at their facility.

**Table 4.** Survey Responses for Facilities That Do Not Offer MOUD (n = 272)

Questions	All facilities, % (95% CI)^a^
Does this clinic specifically screen all patients for opioid use disorder at intake? (n = 272)	
Yes	33.42 (27.06 to 39.79)
No	43.76 (37.03 to 50.49)
Do not know	22.82 (17.11 to 28.52)
Does this clinic plan to start offering medications for opioid use disorder at this location? (n = 272)	
Yes	7.44 (3.96 to 10.91)
No	53.55 (46.79 to 60.31)
Do not know	39.01 (32.39 to 45.63)
Does this clinic refer elsewhere for treatment for opioid use problems? (n = 272)^b^	
Yes	87.28 (82.71 to 91.84)
Among those clinics who refer elsewhere (n = 243)	
When patients are referred to another clinic for medication for opioid use disorder, do they still get counseling or group therapy for their opioid use disorder here at this location?	
Yes	59.71 (52.59 to 66.83)
No	29.71 (23.10 to 36.33)
Do not know	10.58 (6.03 to 15.12)
Is the referral to within same organization or outside of the organization?	
Within same organization	37.51 (30.52 to 44.49)
Outside of the organization	62.49 (55.51 to 69.48)
Among those who refer within the same organization (n = 104)	
What type of clinic is the individual referred to?	
Substance use disorder treatment facility or opioid treatment program	66.98 (55.98 to 77.99)
Mental health clinic	47.91 (36.12 to 59.70)
Health clinic	9.04 (2.37 to 15.70)
Do not know	5.23 (−0.06 to 10.52)
Do you refer these patients to an internal telehealth specialty clinic for treatment?	
Yes	21.83 (12.09 to 31.58)
No	42.92 (31.18 to 54.66)
Do not know	35.25 (24.04 to 46.45)
Among those who refer to an external clinic (n = 139)	
Do you refer these patients to an external telehealth specialty clinic for treatment?	
Yes	19.48 (12.19 to 26.76)
No	51.77 (42.50 to 61.04)
Do not know	28.75 (20.34 to 37.16)
What type of clinic is the individual referred to?	
Substance use treatment clinic	76.84 (69.06 to 84.62)
Health clinic	33.43 (24.69 to 42.17)
Do not know	0.01 (−0.01 to 0.02)

^a^ Sampling weights were used when calculating these frequencies.

^b^ The full question is: “If someone called here looking for mental health treatment as well as medication for a problem with heroin, fentanyl or prescription pain medication, would this clinic (this location/address) refer them somewhere else for treatment for opioid use problems?”

## Discussion

In this study of a representative sample of 450 community outpatient MHTFs in 20 high-burden states, we found less than half offering MOUD. In facilities offering MOUD, buprenorphine (84%) and naltrexone (70%) were most frequently offered, with methadone (14%) offered less commonly, likely because methadone for OUD is highly regulated and available only at opioid treatment programs.^28^

CCBHCs, required to provide integrated SUD treatment, are more likely to offer MOUD than non-CCBHC MHTFs. However, only approximately half of CCBHCs offer MOUD. Notably, offering MOUD on-site is not explicitly required of CCBHCs; the criteria state: “CCBHC staff must include a medically trained behavioral health care provider, either employed or available through formal arrangement, who can prescribe and manage medications independently under state law, including buprenorphine and other FDA-approved medications used to treat opioid, alcohol, and tobacco use disorders.”^29^ Nevertheless, this important requirement implies someone capable of providing MOUD must be available to the clinic, perhaps through referral.

Facilities offering integrated treatment services for people with SUDs, regardless of CCHBC status, are more than 5 times as likely to offer MOUD than facilities without integrated services. Facilities reporting treating people with co-occurring mental health and SUD were more than 2 times as likely. One possible reason is higher patient need and demand: facilities offering integrated services see more clients in need of MOUD. Another possibility is greater acceptability among leadership and clinicians—MHTFs offering a variety of SUD treatment services may be more amenable to offering MOUD. These findings suggest offering integrated SUD services for people with COD as a potential avenue toward improving MOUD access.

Facility-level characteristics associated with an increased probability of offering MOUD include the facility offering housing services and laboratory services. Offering housing services may be seen as an important companion to offering MOUD, as stable housing may facilitate MOUD adherence.^30^ Community outpatient MHTFs offering housing services could be better prepared to initiate MOUD, or facilities with expanded services may simply have greater capacity to offer on-site MOUD. On-site laboratory services may also facilitate expedited MOUD-related lab testing. Finally, while our study was not powered to examine the effect of state funding factors such as Medicaid expansion, the association between state Medicaid expansion and the offering and receipt of MOUD at SUD treatment facilities is well established.^31,32^

Also, important to consider is how MOUD treatment need is addressed in community outpatient MHTFs not offering MOUD on site. More than 87% of facilities reported not offering MOUD, but instead referring patients elsewhere for MOUD, with 38% referring patients to another facility within the same organization. Moreover, when patients are referred elsewhere for MOUD, 60% may still receive OUD-related counseling or group therapy at the referring facility. That more than one-third of MHTFs not offering MOUD refer patients for MOUD to a facility within the same organization suggests the existence of different models of MOUD provision within organizations that may not have the capacity to provide MOUD at all sites. Specifically, this finding could reflect that some of the MHTFs are unable to support a MOUD prescriber, such as lack of institutional support (including protected time, and support in diagnosing and treating patients with OUD),^33^ lack of access to psychosocial services,^34^ and stigma associated with treating OUD and lack of training or comfort prescribing MOUD.^35^ Additional study is needed to better understand variations in models of MOUD provision and how well they meet the need of patients with COD.

### Limitations

This study has limitations. Because we focused on states most affected by the opioid crisis, we do not know if findings generalize to other states. Also, we asked facilities if they offer MOUD, but do not know the extent to which facilities offering MOUD deliver it or if so, how many patients receive it. Previous work has found incongruities between facility reports in the Behavioral Health Treatment Locator and actual practices^36,37^; future work should confirm the extent to which facilities reporting offering MOUD deliver it. Next, we spoke with a clinic call receptionist but did not collect data on the respondent’s occupation. Additionally, we did not gather information about why MHTFs did or did not provide MOUD; more in-depth information is needed to understand the implementation determinants to providing MOUD in these settings.

## Conclusions

Community outpatient MHTFs are an important part of the treatment ecosystem for individuals with co-occurring OUDs. Our study found that approximately one-third of all community outpatient MHTFs and just over half of CCBHCs reported offering MOUD in 20 high-burden states. Importantly, many community outpatient MHTFs that do not offer MOUD on-site indicated that they refer patients to another facility within the same organization. Further attention is needed to address challenges to offering MOUD in MHTFs and to assess whether referral models can effectively meet patients’ needs.

## Notes

Supplement 1. **eAppendix. **Supplemental Materials

Supplement 2.
Data Sharing Statement
