# Supplementary material for: Availability of Medications for Opioid Use Disorder in Community Mental Health Facilities
Source: JAMA Netw Open. 2024 Jun 18;7(6):e2417545. doi: 10.1001/jamanetworkopen.2024.17545 (PMC11185975; doi:10.1001/jamanetworkopen.2024.17545)
Supplement: Supplement 1. — eAppendix. Supplemental Materials [file jamanetwopen-e2417545-s001.pdf]

Supplemental Online Content

Cantor J, Griffin BA, Levitan B, et al. Availability of opioid use disorder medication in community mental health facilities in 20 states. *JAMA Netw Open.* 2024;7(6):e2417545. doi:10.1001/jamanetworkopen.2024.17545

eAppendix. Supplemental Materials

This supplemental material has been provided by the authors to give readers additional information about their work.

© 2024 Cantor J et al. *JAMA Network Open.*

eAppendix. Supplemental Materials

Table of Contents

Supplemental information

Supplemental table 1

Survey protocol and questionnaire

© 2024 Cantor J et al. *JAMA Network Open.*

Supplemental Information

Selection of measures for multivariable regression

Of note, we did not examine all possible facility characteristics available in MATTR in our analysis. Facility level characteristics shown in Table 1 and used in our final logistic regression model were selected purposefully, with only those with a potentially meaningful association with MOUD availability included. First, there are 175 facility-level factors in MATTR that could warrant careful consideration and have yet to be fully explored in the literature, in terms of potential associations with whether or not a MHTF has MOUD available. Thus, we first identified factors upon which our responding facilities were noticeably different using a SMD threshold of 0.1 to denote factors that should be considered (yielding 65 variables). Second, we reviewed the remaining list to determine whether variables not meeting the threshold should or should not be included based on the team's expertise as well as the literature (yielding 39 variables). And, finally, we used correlations to identify any potentially duplicative factors that should be further removed as well removed variables that have small cell sizes to ensure parsimony. This process led to us to identify 25 facility level factors for inclusion in our analyses.

Supplemental Table 1 Descriptive Statistics of Sample

	Successfully Contacted (n=450)		Not Successfully Contacted (n=192)		Total Contacted (n=642)	
	n	%	n	%	n	%
**Certified Community Behavioral Health Clinics**						
Yes	152	33.78	61	31.77	213	33.18
**Payment/Insurance/Funding Accepted**						
State mental health agency (or equivalent) funds	278	61.78	91	47.40	369	57.48
Community Service Block Grants	144	32.00	39	20.31	183	28.50
Community Mental Health Block Grants	202	44.89	65	33.85	267	41.59
State education agency funds	76	16.89	21	10.94	97	15.11
State welfare or child and family services funds	224	49.78	79	41.15	303	47.20
Federal military insurance (e.g. TRICARE)	260	57.78	90	46.88	350	54.52
State corrections or juvenile justice funds	181	40.22	61	31.77	242	37.69
State-financed health insurance plan other than Medicaid	304	67.56	116	60.42	420	65.42
County or local government funds	208	46.22	85	44.27	293	45.64
U.S. Department of VA funds	136	30.22	47	24.48	183	28.50
**Special Programs/Groups Offered**						
Persons with traumatic brain injury (TBI)	67	14.89	11	5.73	78	12.15
Clients with HIV or AIDS	88	19.56	22	11.46	110	17.13
Clients with co-occurring mental and SUDs	320	71.11	104	54.17	424	66.04
**Facility Operation**						
Private non-profit organization	302	67.11	121	63.02	423	65.89
Private for-profit organization	66	14.67	32	16.67	98	15.26
**Facility Vaping Policy**						
Vaping permitted in designated area	169	37.56	59	30.73	228	35.51
**Recovery Support Services**						
Housing services	151	33.56	41	21.35	192	29.91
**Ancillary Services**						
Suicide prevention services	352	78.22	129	67.19	481	74.92
**Testing**						
Laboratory testing	131	29.11	66	34.38	197	30.69
**Facility Smoking Policy**						
Smoking permitted in designated area	186	41.33	64	33.33	250	38.94
**License/Certification/Accreditation**						
Federally Qualified Health Center	59	13.11	21	10.94	80	12.46
**Treatment Approached**						
Integrated Mental and SUD treatment	343	76.22	128	66.67	471	73.36
**County Characteristic**						
Urban	299	66.44	153	79.69	452	70.40
Rural	151	33.56	39	20.31	190	29.60
**State Medicaid Expansion Status**						

© 2024 Cantor J et al. *JAMA Network Open.*

Expanded	339	75.33	139	72.40	478	74.45
Not Expanded	111	24.67	53	27.60	164	25.55
**Census Region**						
Midwest	75	16.67	31	16.15	106	16.51
Northeast	120	26.67	60	31.25	180	28.04
South	188	41.78	74	38.54	262	40.81
West	67	14.89	27	14.06	94	14.64

© 2024 Cantor J et al. *JAMA Network Open.*

Survey Protocol and Questionnaire

IF ASKED: "I'm [NAME], calling from a non-profit research center. We're putting together a report on services for people with opioid use disorder." If they ask what organization, "RAND Corporation."

Hi, I'm calling to find out…

Q1 **Do you offer medication <u>at this location</u> (at this address) for people who have an opioid use disorder alongside a mental health disorder? By medication I mean buprenorphine, such as Suboxone, or methadone or naltrexone, such as Vivitrol.**

(By opioid use disorder I mean they have a problem with heroin, fentanyl, or prescription pain medications.)

(IF DK: Is there someone else there I could ask about this? *IF NO ONE, CODE DK*)

0 NO –> *GO TO Q1a*
1 YES –> *GO TO Q2*
9 DK –> *THANK AND END CALL*
3 NO INTERVIEW POSSIBLE –> *THANK AND END CALL*

Q1a **If someone called here looking for mental health treatment as well as medication for a problem with heroin, fentanyl, or prescription pain medication, would this clinic (this location/address) refer them somewhere else for treatment for opioid use problems?**

(IF DK: Is there anyone else there I could ask about this? *IF NO ONE, CODE DK)*

0 NO –> *GO TO Q1c*
1 YES
9 DK –> *THANK AND END CALL*

Q1b **(Is this provider/Are these providers) you would refer to within the same organization as this clinic, or an external provider (outside of this clinic's organization)?**

0 WITHIN SAME ORGANIZATION –> *GO TO Refer1*
1 EXTERNAL PROVIDER, OUTSIDE OF ORGANIZATION –> *GO TO Refer3*
9 DK –> *THANK AND END CALL*

© 2024 Cantor J et al. *JAMA Network Open.*

Refer1 **(Is this provider/Are these providers) you refer to …**
(CODE ALL THAT APPLY)

0 in another mental health clinic
1 in a substance use treatment clinic, including an opioid treatment program OTP)?
2 in a health clinic, like a primary care clinic?
3 OTHER, SPECIFY: ______________________________________
9 DK

Refer2 **Do you refer patients to a <u>telehealth specialty provider from within the organization for treatment for their opioid use problems</u>? (Telehealth includes video/audio)**

0 NO
1 YES
9 DK

GO TO Refer5

Refer3 **(Is this provider/Are these providers) you refer to**
(CODE ALL THAT APPLY)

1 in a substance use treatment clinic, including an opioid treatment program OTP)?
2 in a health clinic, like a primary care clinic?
3 OTHER, SPECIFY: ______________________________________
9 DK

Refer4 **Do you refer these patients to an external <u>telehealth specialty provider for treatment for their opioid use problems</u>? (Telehealth includes video/audio)**

0 NO
1 YES
9 DK

Refer5 **When patients are referred to another provider for medication for opioid use disorder, do they still get counseling or group therapy for their opioid use disorder <u>here at this location</u>? (For example, dual diagnosis or NA groups.)**

0 NO
1 YES
9 DK

© 2024 Cantor J et al. *JAMA Network Open.*

Note Q1c is the same question as Q5

Q1c **Does this clinic specifically screen all patients for opioid use disorder at intake? That is, asking specifically about problems with opioid use, not just all substances generally.**

0 NO
1 YES
2 OTHER, SPECIFY: ____
9 DK

Q1d **Does this clinic plan to start offering medications for opioid use disorder at this location?**

0 NO –> *GO TO Q1e*
1 YES –> *GO TO Plan1*
9 DK –> *GO TO Q1e*

Plan1 **When will this clinic location start offering medications for opioid use disorder?**

__ __ / __ __ __ __

M M / Y Y Y Y
Enter 9 for DK

Plan2 **Which medications for opioid use disorder will the clinic offer?**
(CODE ALL THAT APPLY, ASK EACH MEDICATION)

1 Buprenorphine (the most common one is Suboxone)
2 Methadone
3 Naltrexone (Vivitrol is injectable, Revia is Oral)
9 DK

Note Q1e is the same question as Q9

Q1e **Is this clinic a Certified Community Behavioral Health Center (CCBHC)? By CCBHC I mean the clinic has been formally designated as a CCBHC by a federal or state agency.**

0 NO
1 YES
9 DK

End **Thank you for your time.**

END CALL

© 2024 Cantor J et al. *JAMA Network Open.*

Q2 **Which medications for opioid use disorder does the clinic offer?**
(CODE ALL THAT APPLY, ASK EACH MEDICATION)

(IF DK TO ALL: Is there someone else there I could ask about this? *IF NO ONE, CODE DK*)

1 Buprenorphine (Most common one is Suboxone)
2 Methadone
3 Naltrexone (Vivitrol is injectable, Revia is Oral)
9 DK –> *THANK AND END CALL*

IF YES TO Buprenorphine, ASK Buptype

Buptype **What types of buprenorphine does the clinic offer?**
(CODE ALL THAT APPLY)

1 Oral/sublingual? (Most common is Suboxone)
2 Injectable? (Sublocade)
3 Implants? (Probuphine)
9 DK

Q3 **How would someone pay for their (buprenorphine/methadone/naltrexone) here?**
(CODE ALL THAT APPLY)

1 Private Insurance
2 Medicaid
3 Medicare
4 Out-of-Pocket (Self-pay/Private pay)
5 OTHER, SPECIFY: ___________________________________
9 DK

Q4 **When is the soonest available intake appointment for someone with a co-occurring mental health and substance use disorder who needs medication treatment for an opioid use disorder?**

ENTER DATE __ __/__ __/__ __
M M / D D/ Y Y
Enter 9 for DK

Date guidelines
"First come-first serve" – enter next possible date
"We're scheduling two weeks out" – enter 2 weeks from today
"Next week" – enter next Monday date
"Middle of next week" – enter next Wednesday date

© 2024 Cantor J et al. *JAMA Network Open.*

Note Q5 is the same question as Q1c

Q5 **Does this clinic specifically screen all patients for opioid use disorder at intake? That is, asking specifically about problems with opioid use, not just all substances generally.**

0 NO
1 YES
2 OTHER, SPECIFY: ____
9 DK

Q6 **What services besides medication are offered at this clinic for people with a co-occurring mental health and opioid use disorder who are on medication for opioid use? Do you offer:** (CODE ALL THAT APPLY)

1 Individual counseling for substance use disorder
2 Group counseling/therapy for substance use disorder
3 Telehealth (audio and/or video) for counseling/therapy visits for opioid use disorder
4 Telehealth (audio and/or video) for visits related to medications for opioid use disorder
5 OTHER, SPECIFY: ____
9 DK

Q7 **How many prescribing providers work at this clinic, either part-time or full-time, and provide any services in-person or via telehealth? By a prescribing provider I mean someone who can prescribe any medication for patients, including a psychiatrist, other physician, Nurse Practitioner, or Physician Assistant.**

ENTER NUMBER OF PROVIDERS: ________
9 DK

Q8 **Are services available in languages other than English at this clinic?**

0 NO
1 YES
9 DK

Note Q9 is the same question as Q1e

Q9 **Is this clinic a Certified Community Behavioral Health Center (CCBHC)? By CCBHC I mean the clinic has been formally designated as a CCBHC by a federal or state agency.**

0 NO
1 YES
9 DK

© 2024 Cantor J et al. *JAMA Network Open.*

Q10 **Is there anything else I should know about treatment for people with co-occurring mental health and opioid-use problems here at this clinic?**

0 NO
1 YES ____ *[max 100 characters]*

End **Thank you for your time.**

END CALL

© 2024 Cantor J et al. *JAMA Network Open.*
