# Supplementary material for: Availability of Medications for Opioid Use Disorder in Community Mental Health Facilities
Source: JAMA Netw Open. 2024 Jun 18;7(6):e2417545. doi: 10.1001/jamanetworkopen.2024.17545 (PMC11185975; doi:10.1001/jamanetworkopen.2024.17545)
Supplement: Supplement 2. — Data Sharing Statement [file jamanetwopen-e2417545-s002.pdf]

Data Sharing Statement

Cantor. Availability of Opioid Use Disorder Medication in Community Mental Health Facilities in 20 States. *JAMA Netw Open*. Published June 18, 2024.
doi:10.1001/jamanetworkopen.2024.17545

Data
Data available: No

Additional Information
Explanation for why data not available: The data can be made available upon request.
